# Proteomic Analysis of Rhesus Macaque Brain Explants Treated With *Borrelia burgdorferi* Identifies Host GAP-43 as a Potential Factor Associated With Lyme Neuroborreliosis

**DOI:** 10.3389/fcimb.2021.647662

**Published:** 2021-06-10

**Authors:** Lianbao Li, Lisha Luo, Taigui Chen, Wenjing Cao, Xin Xu, Yu Zhang, Peng Yue, Yuxin Fan, Jingjing Chen, Meixiao Liu, Mingbiao Ma, Lvyan Tao, Yun Peng, Yan Dong, Bingxue Li, Suyi Luo, Jing Kong, Guozhong Zhou, Shiyuan Wen, Aihua Liu, Fukai Bao

**Affiliations:** ^1^ Department of Microbiology and Immunology, Kunming Medical University, Kunming, China; ^2^ Department of Biochemistry and Molecular Biology, Kunming Medical University, Kunming, China; ^3^ Yunnan Province Key Laboratory of Children’s Major Diseases Research, The Children’s Hospital of Kunming/Kunming Medical University, Kunming, China; ^4^ The Institute for Tropical Medicine, Kunming Medical University, Kunming, China; ^5^ Yunnan Demonstration Base of International Science and Technology Cooperation for Tropical Diseases, Kunming, China

**Keywords:** proteomic analysis, lyme neuroborreliosis, neuroinflammation, HMC3, *Borrelia burgdorferi*, GAP-43

## Abstract

**Background:**

Lyme neuroborreliosis (LNB) is one of the most dangerous manifestations of Lyme disease, but the pathogenesis and inflammatory mechanisms are not fully understood.

**Methods:**

Cultured explants from the frontal cortex of rhesus monkey brain (n=3) were treated with live *Borrelia burgdorferi* (Bb) or phosphate-buffered saline (PBS) for 6, 12, and 24 h. Total protein was collected for sequencing and bioinformatics analysis. In addition, changes in protein expression in the explants over time following Bb treatment were screened.

**Results:**

We identified 1237 differentially expressed proteins (DEPs; fold change ≥1.5 or ≤0.67, *P*-value ≤0.05). One of these, growth-associated protein 43 (GAP-43), was highly expressed at all time points in the explants. The results of the protein-protein interaction network analysis of DEPs suggested that GAP-43 plays a role in the neuroinflammation associated with LNB. In HMC3 cells incubated with live Bb or PBS for 6, 12, and 24 h, real-time PCR and western blot analyses confirmed the increase of GAP-43 mRNA and protein, respectively.

**Conclusions:**

Elevated GAP-43 expression is a potential marker for LNB that may be useful for diagnosis or treatment.

## Introduction

Lyme disease (LD), a zoonosis that was first identified in the United States in the mid-1970s, is caused by the tick-borne bacterium *Borrelia burgdorferi* (Bb); typical symptoms are multiphase and multisystem disorder (Koedel and Pfister, 2017). Erythema migrans is a skin condition that is observed in the early stage of the disease. Without treatment, the treatment can spread to the peripheral nervous system and central nervous system (CNS), leading to Lyme neuroborreliosis (LNB) (Lalor and Segal, 2010; Younger, 2016). In 431 patients with LNB from Denmark, the main manifestations were painful radiculitis (65.9%), cranial palsy (43.4%; primarily facial palsy), and headache (28.3%) (Garcia-Monco and Benach, 2019). Cytokines and chemokines released by immune cells—including those of the nervous system such as microglia (the brain-resident macrophages), astrocytes, and oligodendrocytes (the myelinating cells of the CNS)—have been linked to the neuropathogenesis of LNB (Ramesh et al., 2013). The treatment of LNB is complicated by its multisystem etiology, and prevention and early treatment are critical to optimising outcomes (Bransfield, 2018).

Non-human primates (NHPs) are applicable models for investigating human disease including LD, given their genetic and physiologic similarities and close phylogenetic relationship to humans (Phillips et al., 2014). The rhesus monkey is suitable for investigating the immunologic response to Bb treatment in LNB (Ding et al., 2019) and is presently the only available LNB model.

Microglia detect extracellular changes in the brain and are rapidly activated in response to various noxious stimuli (Morris et al., 2013; Dello Russo et al., 2018). Inappropriate microglia activation has been implicated in several neurologic diseases (Dello Russo et al., 2017; Du et al., 2017). Therefore, we speculated that microglia are involved in the pathogenesis of LNB. Proteomic approaches can be used for protein expression profiling in investigations on LD pathogenesis to identify differentially expressed proteins (DEPs) that can serve as biomarkers or potential drug targets (Kaur et al., 2018; Liu et al., 2018; Savage et al., 2018). In the present study, we used isobaric tags for relative and absolute quantitation (iTRAQ) proteomics technology to assess changes in protein abundance (Peng et al., 2018) in the brain upon spirochetes treatment using frontal cortex (FC) explants from rhesus macaque and cultured cells treated with Bb.

## Materials and Methods

### Animals

Three 1-year-old rhesus macaques (*Macaca mulatta*) of Chinese origin weighing 1.5-3 kg (Monkey-1 and -2, male and Monkey-3, female) were obtained from the Institute of Medical Biology, Chinese Academy of Medical Sciences and Peking Union Medical College [animal permit no. SCXK (DIAN) K20150004]. The animals were healthy and were not treated with Bb. We obtained FC tissue immediately after the animals were euthanized using CO_2_. According to the Guide for the Care and Use of Laboratory Animals and ARRIVE Guidelines for Reporting Animal Research, this experiment was performed. The Animal Ethics and Welfare Committee of Kunming Medical University reviewed and approved this study.

### Preparation of Live Bb

A 1-mL volume of Bb strain 4680 (Germany GSMZ designation of USA ATCC 35210, B31, CIP 102532, please refer to https://www.dsmz.de/collection/catalogue/details/culture/DSM-4680) frozen at −80°C was added to 50 mL of Barbour–Stoenner–Kelly II medium (Ruzić-Sabljić et al., 2006; Zückert, 2007) supplemented with 6% rabbit serum; the mixture was placed in a dark, tightly capped tube at 37°C for seven days. The supernatant was discarded after centrifugation (4°C, 10 min, 2000×*g*) when the cell count was 60–120 per high-power field as determined by dark-field microscopy. After two washes with sterile phosphate-buffered saline (PBS), an appropriate volume of 10% FBS-Roswell Park Memorial Institute (RPMI) 1640 medium with 10% fetal bovine serum (FBS) (Thermo Fisher Scientific, Waltham, MA, USA) for co-culture with FC explants or Minimal Essential Medium (MEM) (Shanghai Zhong Qiao Xin Zhou Biotechnology Co, Shanghai, China) for co-culture with HMC3 human microglia cells was added to a final bacterial concentration of 1×10^7^ bacteria/mL. Our experiments were implemented in the BSL-2 laboratory in Kunming Medical University (Certification No. 2016SW0001).

### FC Explant Co-Culture With Live Bb

**Figure 1:**
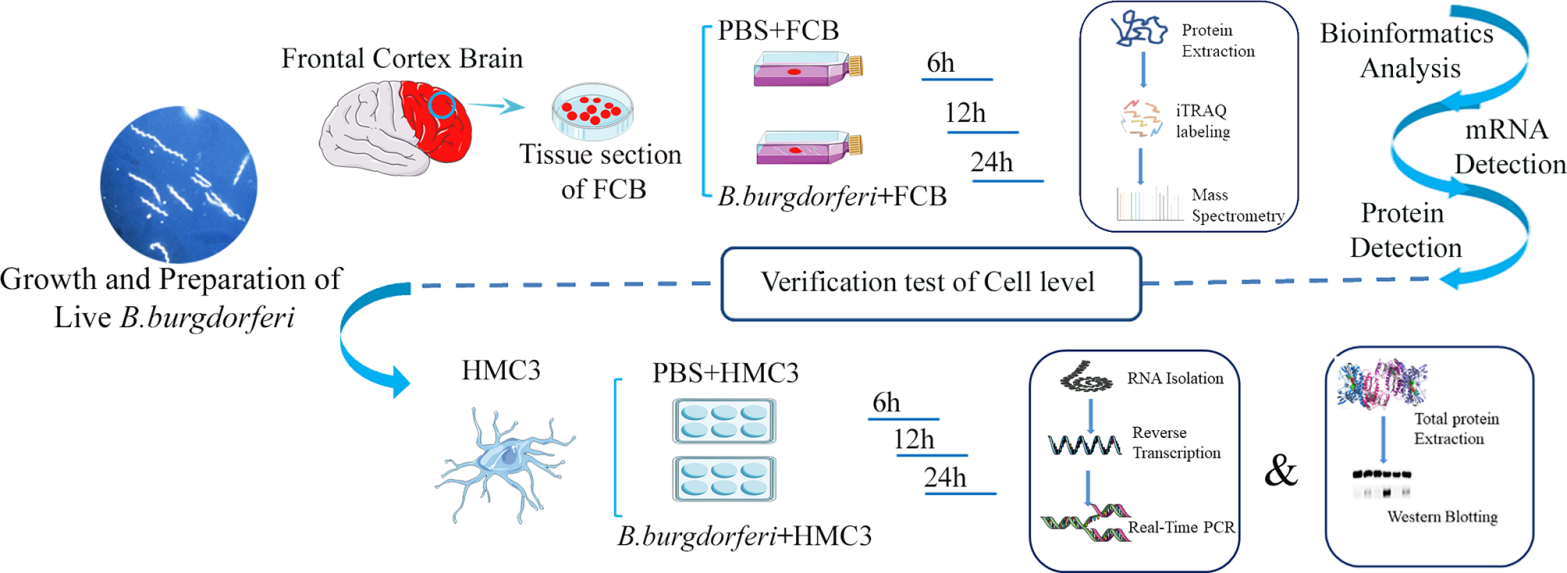
Schematic illustration of the experimental design contains the experiment with explants from the frontal cortex of rhesus macaque brains co-cultured with live Bb and validation experiment using the human microglia HMC3 line.

Fresh FC tissue from 3 rhesus macaques was washed twice with PBS and cut into extremely thin sections using a sterile scalpel [Jinzhong, Shanghai, China; J11060 (handle) and J0B080 (blade)] and atraumatic tissue forceps (Jinzhong; JYF010). Tissue fragments weighing 0.5 g were placed in separate T-25 flasks (Corning, Corning, NY, USA) containing 4 mL of RPMI 1640 medium with 10% FBS or 4 mL Bb suspension prepared as described above. We ensured that each tissue fragment was thoroughly permeated by the medium, and triplicate tissue fragments from the three animals were cultured in a humidified incubator at 37°C and 5% CO_2_ for 6, 12, and 24 h, after which they were collected and stored at -80°C **(** **Figure 1** **)**. We cultured the samples using BSK-II medium during and after the experiment, and no other bacteria were found after bacterial staining and subculture.

### iTRAQ-Based Proteomic Analysis

#### Protein Extraction and Sample Preparation

FC/Bb samples were added to protein lysis solution [7 M urea, 2 M thiourea, 4% sodium dodecyl sulfate, 40 mM Tris-HCl (pH 8.5), 1 mM phenylmethane sulfonyl fluoride (PMSF), and 2 mM EDTA] and incubated on ice for 5 min. Dithiothreitol (DTT) was added to a final concentration of 10 mM, followed by ultrasonication for 15 min in ice. The supernatant was centrifuged (4°C, 10 min, 13,000×*g*) and cold acetone was added, followed by overnight incubation at -20°C.

The protein precipitate was collected by centrifugation and air-dried. After adding a solution of 8 M urea and 100 mM triethylammonium bicarbonate (TEAB) (pH 8.0) to redissolve the proteins, DTT was added to a final concentration of 10 mM and the reduction reaction was carried out in a water bath at 56°C for 30 min. Iodoacetamide was added at a final concentration of 55 mM and the mixture was incubated at room temperature for 30 min in the dark for the alkylation reaction. The Bradford assay was used to determine the protein concentration, and 100 μg of protein from each sample was used for trypsin digestion. After diluting the protein solution 1:4 with 100 mM TEAB, trypsin was added at trypsin:protein mass ratio of 1:50 followed by enzymatic hydrolysis overnight at 37°C. The resultant peptides were desalted and freeze-dried under vacuum.

#### iTRAQ Labeling and Mass Spectrometry

iTRAQ-8 labelling was performed using a commercial kit (Sciex, Redwood City, CA, USA) according to the manufacturer’s instructions. Briefly, peptides were dissolved in 0.5 M TEAB and labelled and mixed. The Ultimate 3000 high-performance liquid chromatography system (Dionex, Thermo Fisher Scientific, USA) was used for fractionation, and the 42 subfractions were combined into 12 samples that were desalted and vacuum-dried on a Strata-X column and dissolved in a solution of 2% acetonitrile and 0.1% formic acid. Mass spectrometry (MS) analysis was performed using a TripleTOF 5600+ instrument coupled with the Eksigent nanoLC system (Sciex, USA).

The peptide solution was added to a C18 capture column (5μm, 100μm×20mm), and gradient elution was performed on a C18 analytical column (3μm, 75μm×150mm) with a mobile phases A (2% acetonitrile, 0.1% formic acid and 98% H_2_O) and a mobile phases B (98% acetonitrile, 0.1% formic acid and 2% H_2_O) at a flow rate of 300 nL/min. The iTRAQ-based proteomics data were acquired using Information Dependent Acquisition (IDA), the MS spectrum was scanned with an ion accumulation time of 250ms and 50 ms automated MS/MS product ion scans were performed for the top-30 ions with the highest intensity. IDA parameters were set as follows: collect MS1 spectrum in the range of 350-1500m/z, collect MS2 spectrum in the range of 100-1500m/z and the dynamic exclusion time was 15 s.

### Bioinformatics Analysis

After optimization processing, tandem MS data were analyzed using the Proteinpilot v4.5 search engine, which is aligned to the TripleTOF 5600+ system. Only peptides with unused score ≥1.3 (reliability level >95%) were retained for protein quantification. DEPs were identified based on the ratios of differently labeled proteins and P values in Proteinpilot. Fold change was calculated as an average value from paired comparisons among biological replicates. We set restrictive conditions in order to identify proteins involved in nervous system damage; only those with fold change in expression ≤0.67 or ≥1.5 and *P*-value <0.05 for differences in protein abundance in all pairwise comparisons were identified as DEPs. To determine the function and biological significance of identified proteins, a homology search was performed in the UniProt database for rhesus macaque (https://www.uniprot.org/), and homologous proteins were subjected to Gene Ontology (GO; http://geneontology.org/), Clusters of Orthologous Genes (COG; http://www.ncbi.nlm.nih.gov/COG/); and Kyoto Encyclopedia of Genes and Genomes (KEGG) pathway (https://www.kegg.jp/kegg/pathway.html) analyses. We also performed functional enrichment (GO and KEGG pathway) analyses for significantly different proteins.

### HMC3 Cells Co-Culture With Live Bb

HMC3 cells (Shanghai Zhong Qiao Xin Zhou Biotechnology Co, Shanghai, China) were cultured in MEM with 5% cattle serum and 1% penicillin/streptomycin (Sangon Biotech, Shanghai, China) at 37°C and 5% CO_2_. The cells were seeded in a 6-well plate at 2.5×10^5^ cells/mL. After 12 h, the supernatant was discarded when the cells fully adhered to the plate. The cells were stimulated with 2.5×10^6^ bacteria/mL Bb, and the PBS only condition was used as a negative control for 6, 12, and 24 h. When preparing the 2.5×10^6^ bacteria/mL Bb suspension, we first diluted Bb to 1×10^9^ bacteria/mL with PBS and then further diluted to the target concentration using 10% FBS-MEM medium. At this point, the original Bb solution was diluted 400 times, so we chose a PBS concentration of 0.25% when setting up PBS as the control group.Cell lysates were prepared using RNAiso Plus reagent (Takara Bio, Otsu, Japan) and stored at -80°C until RNA extraction. Protein lysates were prepared using PMSF and radioimmunoprecipitation assay (RIPA) buffer (Solaibao Life Science, Beijing, China) and stored at -80°C until western blot analysis **(** **Figure 1** **)**.

### RNA Isolation and Reverse Transcription

RNAiso Plus reagent (Takara Bio) was used to extract total RNA from 20 g of brain tissue; chloroform–isopropanol extraction was performed to obtain RNA from HMC3 cells. After incubation for 30 min, the samples were centrifuged and isolated RNA was washed with 75% ethanol. RNA concentration and purity were determined with a Nanodrop-2000 spectrophotometer (Thermo Fisher Scientific), and the approved samples [optical density at 260 nm (OD260) <1.7 and OD280 <2.0] were stored at -80°C. Contaminating DNA was removed by DNase treatment; the RNA was digested at room temperature in 5× gDNA Eraser buffer (Takara Bio) for 5 min, and then reverse-transcribed into cDNA using the PrimeScript cDNA RT kit (Takara Bio). The reaction mixture contained the following: 1 μg RNA, 4 μL 5× PrimeScript Buffer 2 (for real-time PCR), 1 μL PrimeScript RT Enzyme Mix I, 1 μL RT Primer Mix, and RNase dH2O in a final volume of 20 μL. The reaction was performed on a C1000 Touch thermal cycler (Bio-Rad, Hercules, CA, USA) under the following conditions: 37°C for 15 min, 85°C for 5 s, and 4°C for an unlimited duration. The cDNA samples were stored at -20°C.

### Quantitative Real-Time (qRT-)PCR Analysis of Growth-Associated Protein 43 (GAP-43) mRNA Expression in FC Tissue and HMC3 Cells

q-PCR was performed using SYBR R Premix Ex Taq II (Tli RNaseH Plus, 2×) (Takara Bio) on a CFX96 Real-Time PCR system (Bio-Rad) according to the manufacturer’s protocol. The reaction volume was 25 μL. Relative gene expression levels were calculated with the comparative cycle threshold method with normalization to the housekeeping gene glyceraldehyde 3-phosphate dehydrogenase (GAPDH). The following forward and reverse primers were used for amplification: human GAP-43, 5′-GCTCTGAATTATGCCACCCC-3′ and 5′-AGGTCGAACTGCTCTCTGAA-3′; rhesus macaque GAP-43, 5′-AAGAAGCGAGAGGGTGATGC-3′ and 5′-GTGGCACTTTCTGTCTCAGC-3′; human GAPDH, 5′-TGAGAACGGGAAGCTTGTCA-3′ and 5′- ATCGCCCCACTTGATTTTGG-3′; and rhesus macaque GAPDH, 5′-GCACCACCAACTGCTTAGCAC-3′ and 5′-TCTTCTGGTGGCAGTGATG-3′.

### Western Blot Analysis of GAP-43 Protein Expression in FC Tissue and HMC3 Cells

Total protein was extracted from FC tissue and HMC3 cells using PMSF and RIPA buffer and quantified with the bicinchoninic acid method. The western V3 workflow (Bio-Rad) was used for western blot analysis. The primary antibodies [anti-GAP-43 (ab12274 and ab11136) and anti-GAPDH (ab181602)] and secondary antibody [horseradish peroxidase-conjugated goat anti-rabbit IgG H&L (ab6721)] were purchased from Abcam (Shanghai, China).

## Results

### Quality Control for Proteomics Analysis

The experimental protocol is illustrated in **Figure 1**. We identified 26,493 and 26,309 peptides in 2 independent iTRAQ experiments, corresponding to 3598 and 3590 proteins, respectively. We judged whether the identified proteins were reliable based on the unique peptides, which only exist in one protein. The number of proteins containing at least 2 unique peptides in the 2 datasets was 2909 and 2918, accounting for 80.85% and 81.28% of total proteins, respectively **(** **Supplementary Figure 1A** **)**. As each mass spectrometer has a defined measurement range, the peptides that can be identified have a length limit. The identified peptides had lengths between 7 and 20, with a mode of 11 and a mean of 13.98, which was within a reasonable range **(** **Supplementary Figure 1B** **)**. A greater number of peptides increases the reliability of an identified protein; thus, the coverage of identified proteins indirectly reflects the overall accuracy of the results. The percentage of proteins with coverage of 0–10% was 38.49%; the average protein coverage was 20.76%, and proteins with coverage ≥20% accounted for 39.28% of total proteins **(** **Supplementary Figure 1C** **)**. These results demonstrate that our iTRAQ results were relatively reliable.

### Identification and Function Annotation of DEPs

We used 3 different databases (GO, KEGG pathway, and COG) to predict the functions and biological significance of proteins identified by iTRAQ analysis for LNB. Not all of the 4450 identified proteins were annotated in the databases; 4321, 2339, and 2642 proteins were annotated in the GO, COG, and KEGG databases, respectively.

The identified proteins were divided into 3 GO functional classes (Biological Process, Cellular Component, and Molecular Function), and subcategorised into 58 hierarchical GO classifications for detailed investigation **(** **Supplementary Figures 1D–F** **)**. We performed independent functional annotation analyses on proteins that were changed over time in FC/Bb co-cultures. For example, there were 368 DEPs (299 increased and 69 decreased) in FC samples cultured with Bb as compared to PBS for 24 h (Bb-24h and P-24h, respectively); a comparison of GO annotation results revealed major differences in GO functional classifications between changed proteins **(** **Supplementary Figure 2A** **)**. For example, the function “Protein-binding transcription factor activity” was associated with the former but not the latter set of proteins. We also compared the identified proteins with the COG database, predicted the possible functions of these proteins and made classification statistics. The COG annotations for the identified proteins are shown in **Supplementary Figure 2B**.

Pathway-based analyses can identify the main biochemical, metabolic, and signal transduction pathways in which a protein is involved. We performed the KEGG pathway and functional enrichment analyses of the DEPs **(** **Supplementary Figures 2C–E** **)**. Bb treatment had a marked effect on the functional profile of the FC of rhesus macaque, as evidenced by the fact that the DEPs were mainly associated with the pathogenesis of Parkinson’s disease, Huntington disease, Alzheimer’s disease, and signalling pathways related to neural function **(** **Supplementary Tables 1**, **2** **)**.

### Analysis of DEPs

**Figure 2:**
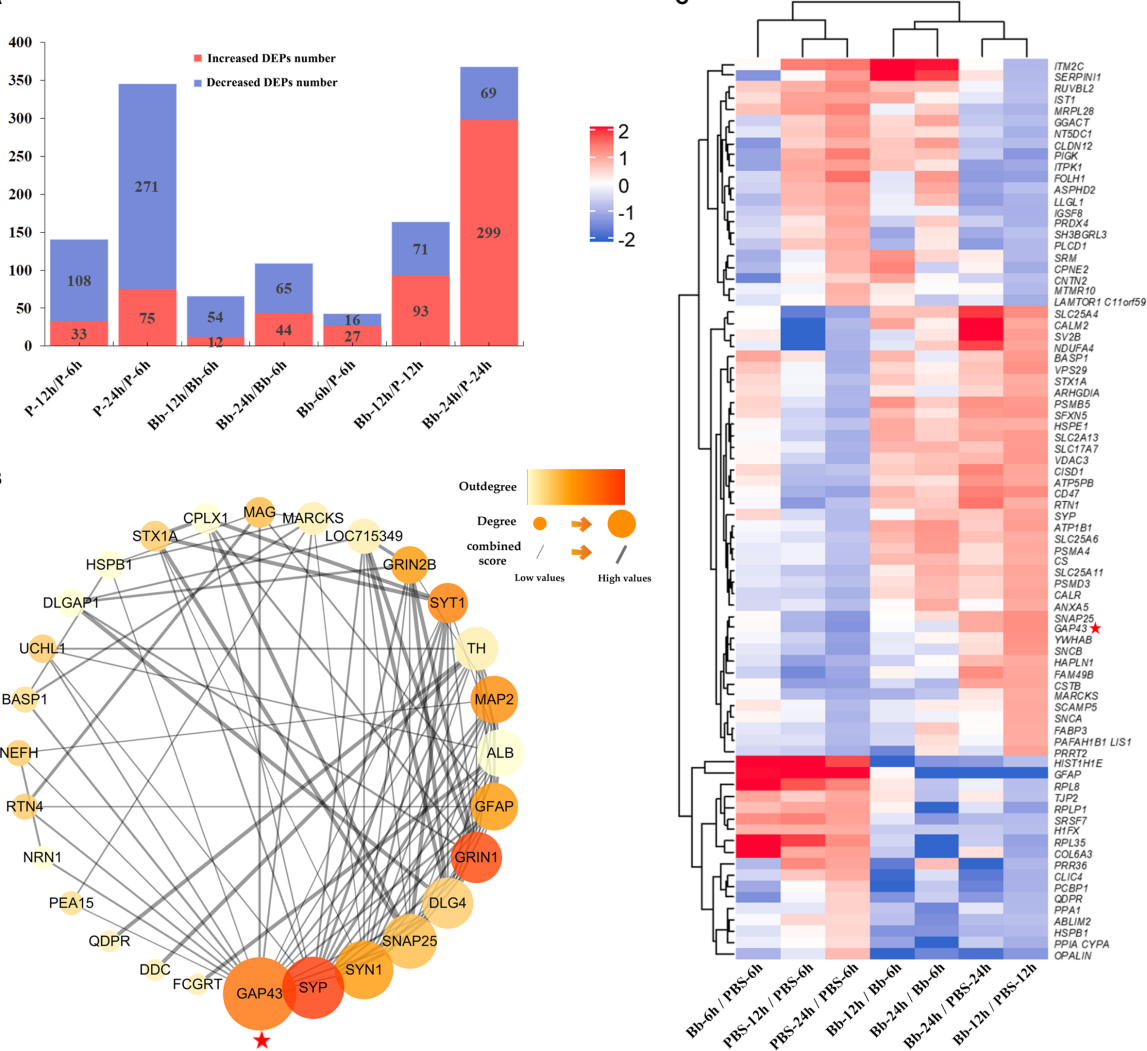
Screening of DEPs in this study. **(A)** The number of increased and decreased DEPs in different groups. **(B)** Heatmap of DEPs identified by protein sequencing showing the hierarchical clustering of the relative expression of a portion of the DEPs in each group for brevity. High and low abundance of protein expression is shown in red and blue, respectively. **(C)** Analysis of our target protein, GAP-43, by PPI network to find its related proteins for the following study. Our target protein has been marked with red pentagrams in the figure.

We analysed differences in protein expression levels between the Bb-treated and control (PBS) samples (fold change ≥1.5 or ≤0.67, *P ≤* 0.05) (Liu et al., 2013). Our laboratory also performed a combined transcriptomic and proteomic analysis in the early stage (Ding et al., 2020). The number of DEPs between the experimental and control groups increased over time **(** **Figure 2A** **)**; a heatmap of the DEPs is shown in **Figure 2B**, and the top 10 changed proteins in each group are shown in **Supplementary Table 3**. GAP-43 level decreased over time in the PBS/Bb-treated FC explants, but the level was significantly greater in the Bb group compared with the PBS group at 24 h time point (fold change =24.59, *P*=0.012).

To investigate the interactions of proteins in the FC in response to Bb treatment, we performed a protein-protein interaction (PPI) network analysis using the STRING web resource (https://string-preview.org/) and Cytoscape v3.8.1 software. The PPI network contained 28 nodes and 101 edges and revealed network crosstalk between GAP-43 and related DEPs **(** **Figure 2C** **)**. We selected GAP-43 for more detailed analysis as it was located upstream of the PPI network.

### Validation of GAP-43 mRNA and Protein Expression in FC Explants Stimulated With Bb

**Figure 3:**
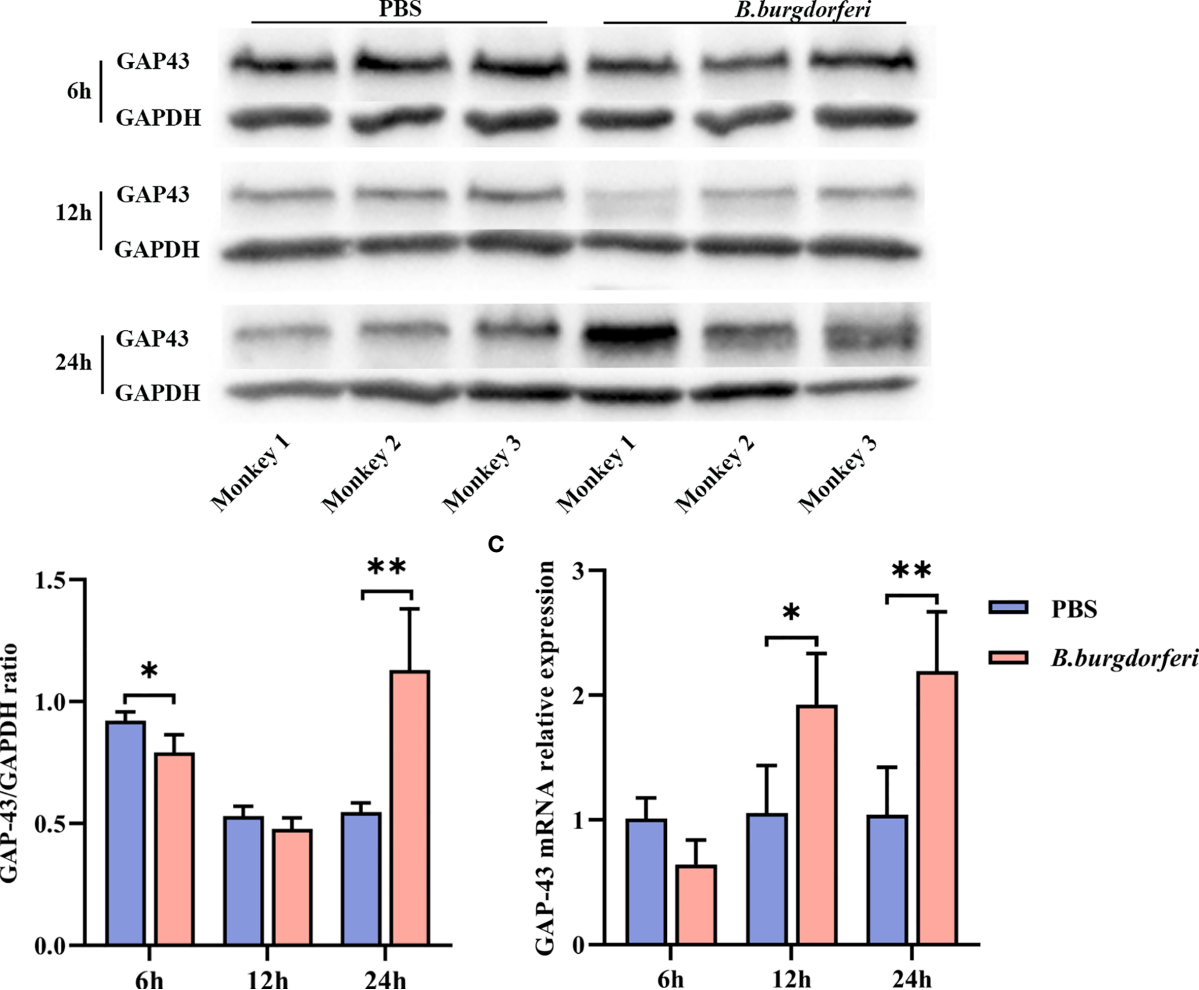
Significant alteration of GAP-43 mRNA and protein levels in explants from the frontal cortex of rhesus macaque brains at different time points after Bb treatment. **(A)** Protein expression of GAP-43 in explants from the frontal cortex of rhesus macaque brains co-cultured with live Bb and the controls at 6, 12, and 24 h, validated by western blotting. **(B)** Quantitative analysis of GAP-43 protein expression levels. **(C)** qPCR analysis of GAP-43 mRNA expression comparing explants from the frontal cortex of rhesus macaque brains co-cultured with live Bb with the controls at 6, 12, and 24 h. **P < 0.01, *P < 0.05. Analyzed with two-way ANOVA and data were expressed as the means ± SD.

We examined GAP-43 mRNA and protein expression in rhesus monkey FC explants stimulated with Bb by qRT-PCR and western blot, respectively. There was no significant difference in GAP-43 protein level in FC explants treated with Bb for 12 h relative to the PBS control; however, at 24 h, GAP-43 level was higher in the Bb treatment group **(** **Figures 3A, B** **)**. Additionally, GAP-43 mRNA level was higher in the Bb group than in the PBS group at 12 and 24 h, which is consistent with the results of the proteomics analysis **(** **Figure 3C** **)**.

### Validation of GAP-43 mRNA and Protein Expression in HMC3 Cells Stimulated With Bb

**Figure 4:**
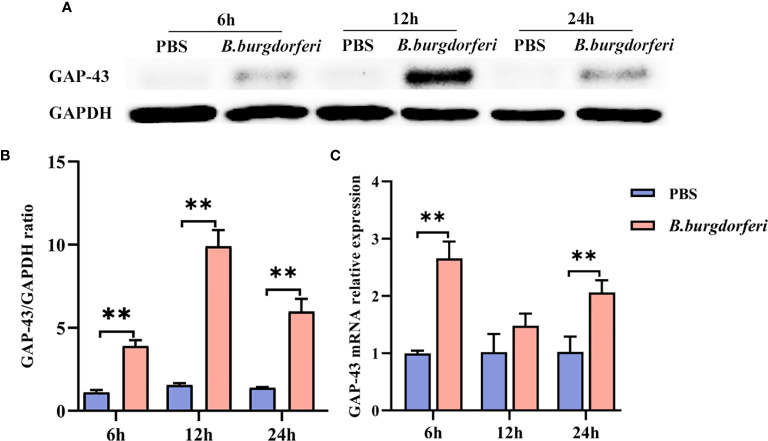
Significant alteration of GAP-43 mRNA and protein levels after Bb treatment with HMC3. **(A)** Protein expression of GAP-43 in HMC3 cell lines, co-cultured with live Bb and controls at 6, 12 and 24 h, was verified by western blotting. **(B)** Quantitative analysis of GAP-43 protein expression levels. **(C)** qPCR analysis of GAP-43 expression in HMC3 cell lines, comparing co-cultured with live Bb and controls at 6, 12 and 24 h. **P < 0.01. Our data were analyzed with two-way ANOVA and expressed as the means ± SD.

We investigated whether Bb affects GAP-43 mRNA and protein expression in human microglia using HMC3 cells at 6, 12, and 24 h after treatment. At all three time points, GAP-43 protein **(** **Figures 4A, B** **)** and mRNA **(** **Figure 4C** **)** expression was increased in HMC3 cells treated with Bb compared to those treated with PBS.

## Discussion

LD is a natural disease caused by treatment with Bb (Shapiro, 2014), which uses various strategies to overcome the host’s innate and adaptive immune responses and induces systemic symptoms (de Taeye et al., 2013) including neurologic dysfunction (Rupprecht et al., 2008). Treatment of the brain is considered as the most dangerous manifestation of LD. In order to gain insight into the mechanism by which Bb causes neurologic damage, we carried out proteome profiling of rhesus macaque FC explants treated with Bb, and verified the findings using a human microglia cell line. Spirochetes have been detected at nerve roots in a rhesus macaque model of LNB (Pachner et al., 2001). The HMC3 cell line was established through SV40-dependent immortalisation of human embryonic microglia and have the ability to grow rapidly (proliferating between 24-48 h) and retain most of the phenotypic and morphological characteristics of primary microglia, recently authenticated by the American Type Culture Collection (ATCC CRL-3304; Manassas, VA, USA. Please refer to https://www.atcc.org/products/all/CRL-3304.aspx), and has been used in several studies (Dello Russo et al., 2018; Angel-Ambrocio et al., 2020; Hankittichai et al., 2020). As macrophages in the brain, microglia cells have many functions such as phagocytosis, antigen presentation and cytokine secretion, and play an important role in many degenerative neurological diseases (Perry et al., 1993; Janabi et al., 1995).

Our analyses demonstrated that Bb treatment altered the proteomic landscape of the brain in an NHP. More specifically, GAP-43 was among many proteins that were differentially expressed between FC explants or HMC3 cells exposed to Bb *vs* PBS; at each examined time point, the mRNA and protein levels of GAP-43 were increased in the Bb-treated samples. GAP-43 is mainly expressed in the endoolfactory cortex, neocortex, and hippocampus, and is anchored on the cytoplasmic side of the presynaptic plasma membrane where it regulates synaptic plasticity and regenerative axonal growth and plays a critical role in neural development, axonal outgrowth, and stabilisation of synaptic function (Holahan, 2017). GAP-43 was shown to promote neurite outgrowth during development and nervous system repair by inducing F-actin accumulation, stimulating morphogenic activity, and preventing growth cone retraction (Zhang et al., 2018; Camporesi et al., 2020); changes in GAP-43 expression have been reported following nerve injury and in CNS diseases such as stroke, Alzheimer disease, epilepsy, and Parkinson disease (Tönges et al., 2014; Nemes et al., 2017; Sandelius et al., 2018; Sandelius et al., 2019; Tible et al., 2020).

Like many bioactive proteins, GAP‐43 is intrinsically disordered, lacking a stable tertiary and/or secondary structure under *in vitro* physiologic conditions (Uversky, 2010; Forsova and Zakharov, 2016), the functional significance of this feature has not been thoroughly studied (Chiti and Dobson, 2006). Nonetheless, many disordered proteins such as amyloid β-protein and tau protein are soluble or can form aggregates that have been linked to the pathogenesis of neurodegenerative diseases (Caminati and Procacci, 2020). GAP-43 functions as an actin regulator and changes in its expression can lead to accumulation of the actin-depolymerising factor cofilin and the formation of cofilin–actin rods, which can impair synaptic function and cause the loss of synapses (Chung et al., 2020). Nerve damage stimulates the release of cytokines and neurotrophic factors by neurons and glia and activates growth-related proteins such as GAP-43, thereby enhancing neuroprotection and regeneration (Filbin, 2003; Silver and Miller, 2004). Given its dual functions in the CNS, it remains unclear whether the observed increase of GAP-43 in LNB is pathogenic or protective.

Bb induces the production of inflammatory mediators in the CNS, which is accompanied by neuronal and/or glial apoptosis (Ramesh et al., 2008; Ramesh et al., 2015). We previously analysed the transcriptome profile of rhesus macaques brain preFC explants following Bb treatment; the results showed that genes related to the immune response were significantly increased including interleukin 6 (IL-6) signal transducer (*IL6ST*), colony-stimulating factor 1 receptor (*CSF1R*), C1q and tumour necrosis factor-related 7 (*C1QTNF7*), CX3C chemokine receptor 1 (*CX3CR1*), and C-C motif chemokine ligand 24 (*CCL24*) (Ding et al., 2019). While most neurons in the CNS of adult mammals cannot regenerate axons after injury, increased levels of IL-6 can activate the phosphatidylinositol 3-kinase (PI3K)/protein kinase B (Akt) and Janus kinase (JAK)/signal transducer and activator of transcription 3 (STAT3) signalling pathways, resulting in the increased of GAP-43 and other growth factors and consequently, neurite outgrowth and synapse formation (Kossmann et al., 1996; Yang et al., 2012). Whether this mechanism applies to the repair of nerve damage caused by LNB remains to be determined. Our PPI network analysis revealed several other proteins besides GAP-43 that were increased in FC explants by Bb treatment and can potentially mediate neuronal repair including synaptophysin (SYP), brain acid-soluble protein 1 (BASP1), and synapsin-1 (SYN-1).

Given the severity of clinical symptoms and poor prognosis of LNB, there is an urgent need to establish the pathogenic mechanisms and identify predictive biomarkers and therapeutic targets. Our study demonstrated that treatment of FC explants from an NHP with Bb—the causative agent of LNB—altered the expression of many proteins including GAP-43. Although these findings are preliminary and must be verified in additional studies, they provide a basis for further exploration of the pathogenesis of LNB and indicate that therapeutic targeting of GAP-43 may be a promising strategy for its treatment. However, the use of NHPs for research is limited by ethical considerations and high cost; these can be circumvented by using human brain organoids as experimental models. In recent years, some scientists have started to use human brain organoids to replace animal models to study infectious diseases (Xu et al., 2019; McMahon et al., 2021). In the future, we also use human brain organoids to replace animal models to study Lyme neuroborreliosis.

## Data Availability Statement

The datasets presented in this study can be found in online repositories. The names of the repository/repositories and accession number(s) can be found in the article/**Supplementary Material**.

## Ethics Statement

The animal study was reviewed and approved by The Animal Ethics and Welfare Committee of Kunming Medical University.

## Author Contributions

FB, AL, LBL, LSL, and TC conceived and designed the experiments. LBL, LSL, TC, MM, LT, and YP developed the methodology. LBL, LSL, TC, YZ, XX, WC, PY, YF, ML, and JC performed all experiments. LBL, LSL, TC, YD, JK, SL, BL, SW, and GZ analyzed and discussed the data. LBL, LSL and TC wrote the manuscript. LBL, LSL, TC, FB, and AL edited and revised the manuscript. All authors contributed to the article and approved the submitted version.

## Funding

This work was supported by grants from the National Natural Science Foundation of China (No. 32060180, 81860644, 81560596, and 31560051) and the Natural Foundation of Yunnan Province [No. 2019FE001(-002) and 2017FE467(-001)]. The funding institutions had no involvement in the design of the study or review of the manuscript.

## Conflict of Interest

The authors declare that the research was conducted in the absence of any commercial or financial relationships that could be construed as a potential conflict of interest.
