# Supplementary figures and images for: Proteomic Analysis of Rhesus Macaque Brain Explants Treated With Borrelia burgdorferi Identifies Host GAP-43 as a Potential Factor Associated With Lyme Neuroborreliosis
Source: Front Cell Infect Microbiol. 2021 Jun 10;11:647662. doi: 10.3389/fcimb.2021.647662 (PMC8224226; doi:10.3389/fcimb.2021.647662)

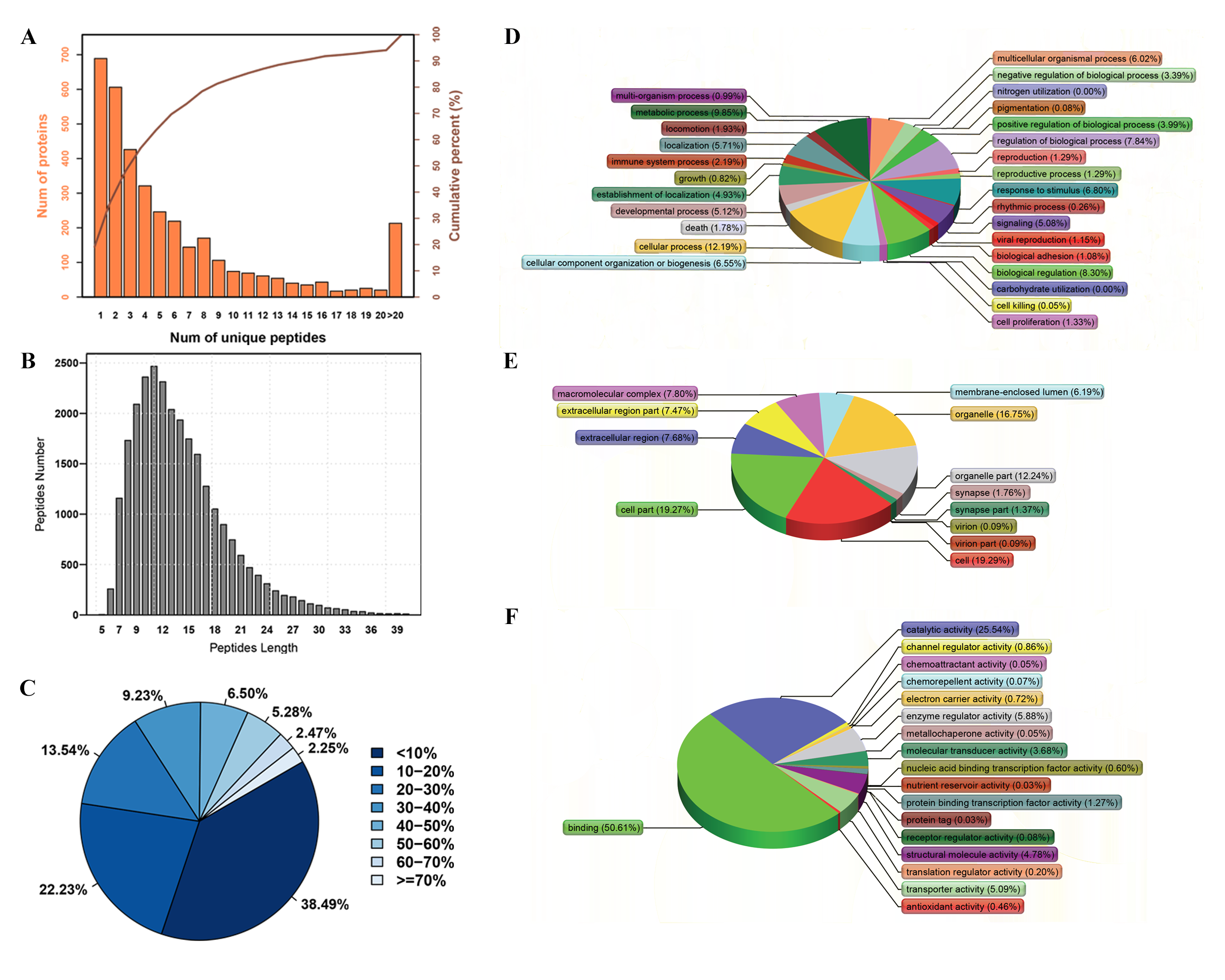

Supplement: Supplementary file 1 [file Image_1.tif]

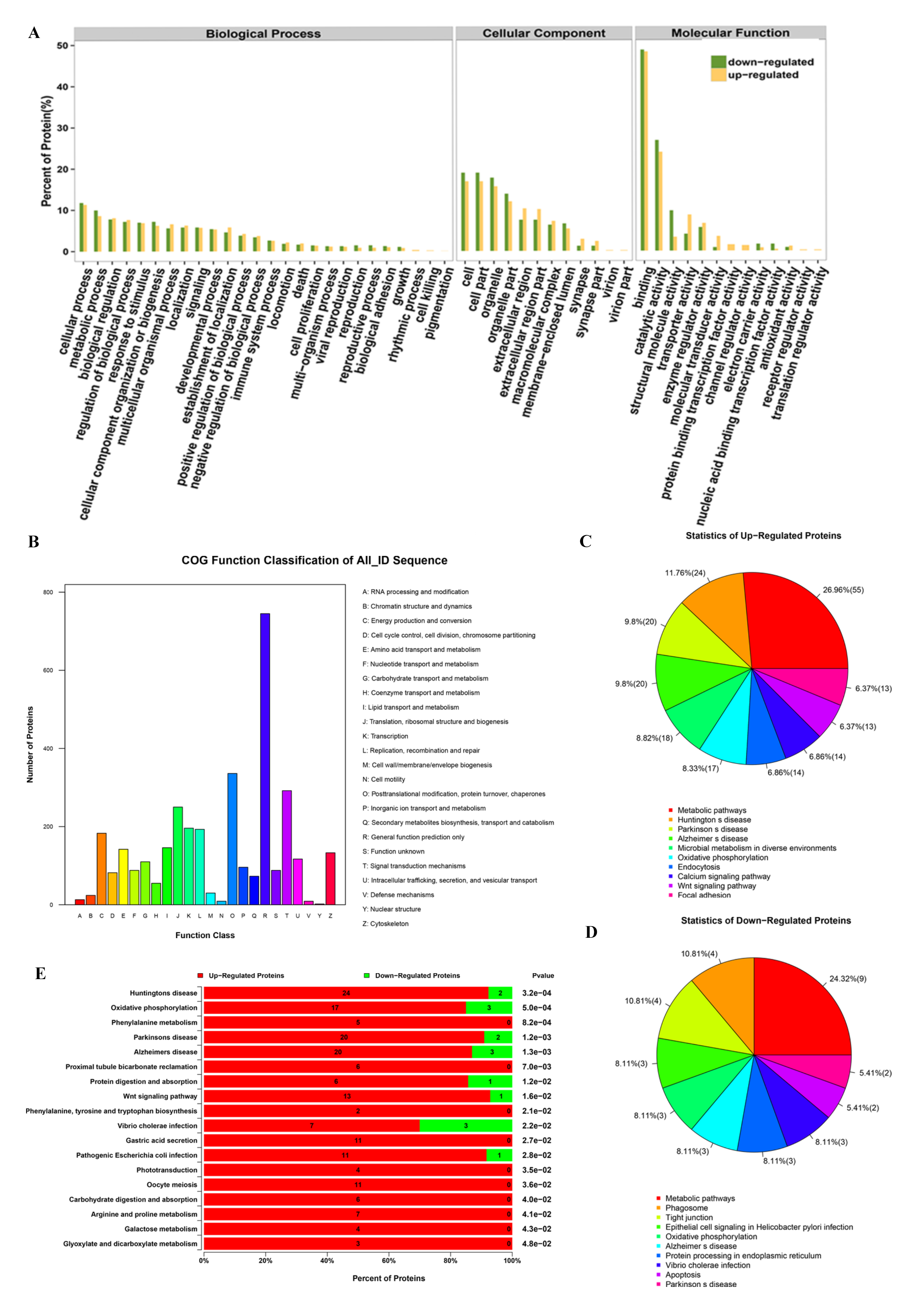

Supplement: Supplementary file 2 [file Image_2.tif]
