# Supplementary material for: Proteomic Analysis of Rhesus Macaque Brain Explants Treated With Borrelia burgdorferi Identifies Host GAP-43 as a Potential Factor Associated With Lyme Neuroborreliosis
Source: Front Cell Infect Microbiol. 2021 Jun 10;11:647662. doi: 10.3389/fcimb.2021.647662 (PMC8224226; doi:10.3389/fcimb.2021.647662)
Supplement: Supplementary file 3 [file DataSheet_1.docx]

Supplementary Table 1: GO enrichment of differentially expressed proteins (DEPs) between different groups.

Supplementary Table 2: Pathway enrichment of differentially expressed proteins (DEPs) between different groups.

Supplementary Table 3: Screening of differentially expressed proteins (DEPs) by differential ploidy and related research pathways.

Supplementary Figure 1：Quality control of proteomics analysis and quantification of differentially expressed proteins (DEPs) in our study. (A). The number of unique peptides that can uniquely determine the presence of a protein of the identified proteins. (B). Distribution of peptide lengths after proteolytic digestion using appropriate proteases. (C). Coverage of identified proteins. The different colored pies in the distribution pie chart represent the percentage of proteins with different ranges of identification coverage. (D-F). GO classification of all quantified proteins in Biological Process, Cellular Component, and Molecular Function.

Supplementary Figure 2：Functional and pathway analysis of differentially expressed proteins (DEPs) in our study. (A). GO classification of DEPs in the 3 domains in the Bb-24h/P-24h group. The yellow bar is the proportion of functional classification under the three ontologies of up-regulated differential proteins in the Bb-24h/P-24h group. The green bar is the proportion of functional classification under the three ontologies of down-regulated differential proteins. (B). COG functional classification of all quantified proteins predicting the possible functions of these proteins and classifying them into functional statistics. (C-D). Pathway metabolic pathway annotation in the Bb-24h/P-24h group. The number of proteins under each function is indicated in parentheses. (E). Pathway enrichment analysis of DEPs in the Bb-24h/P-24h group. Red indicates proteins with up-regulated expression. The figure reflects the distribution of significant differential proteins under different functional pathways, where the right side represents the P-value of the significance level enriched in this pathway, the red and green areas of the bar are the percentages of identified up-regulated and down-regulated proteins contained in this pathway, respectively, and the numbers on the bar represent the number of corresponding differential proteins.
